# Performance of Computed Tomography Angiography (CTA) for the Diagnosis of Hypo-Attenuated Leaflet Thickening (HALT)

**DOI:** 10.3390/jcm11071817

**Published:** 2022-03-25

**Authors:** Manuel Hein, Philipp Breitbart, Jan Minners, Philipp Blanke, Simon Schoechlin, Christopher Schlett, Tobias Krauss, Martin Soschynski, Franz-Josef Neumann, Philipp Ruile

**Affiliations:** 1 Department of Cardiology & Angiology II, University Heart Center Freiburg, 79106 Freiburg, Germany; philipp.breitbart@uniklinik-freiburg.de (P.B.); jan.minners@uniklinik-freiburg.de (J.M.); simon.schoechlin@uniklinik-freiburg.de (S.S.); franz-josef.neumann@uniklinik-freiburg.de (F.-J.N.); philipp.ruile@uniklinik-freiburg.de (P.R.); 2 Department of Radiology, St. Paul’s Hospital, University of British Columbia, Vancouver, BC V6Z1Y6, Canada; phil.blanke@gmail.com; 3 Department of Diagnostic and Interventional Radiology, Medical Center-University of Freiburg, Faculty of Medicine, University of Freiburg, 79106 Freiburg, Germany; christopher.schlett@uniklinik-freiburg.de (C.S.); tobias.krauss@uniklinik-freiburg.de (T.K.); martin.soschynski@uniklinik-freiburg.de (M.S.)

**Keywords:** TAVR, TAVI, hypo-attenuated leaflet thickening, leaflet thrombosis, post-TAVR-CTA, computed tomography

## Abstract

(1) Background: Early hypo-attenuated leaflet thickening (HALT) is diagnosed by computed tomography angiography (CTA) in approximately 15% of patients undergoing transcatheter aortic valve replacement (TAVR). We sought to investigate the diagnostic performance of CTA for the diagnosis of HALT, focusing on timing data assessment within the cardiac cycle. (2) Methods: The study enrolled 50 patients with and 50 without HALT with available post-TAVR-CTA. The primary objective was to compare the diagnostic performance of CTA readings at specific intervals and time points during the cardiac cycle (entire systole, entire diastole, end-systole, and mid-diastole) versus gold standard (consensus reading by two observers based on multiphase full cardiac cycle data sets). (3) Results: 100 CTAs were independently analysed by two observers blinded to clinical characteristics of the study population and the results from the gold standard reading. Sensitivity and specificity for the diagnosis of HALT were 84%/94% in systole, 87%/92% in diastole, 78%/95% at end-systole, and 80%/94% at mid-diastole. End-systole had the highest positive predictive value (0.88) and positive likelihood ratio (36). Cohen’s kappa for interobserver reliability was 0.715 in systole, 0.578 in diastole, 0.650 at end-systole, and 0.517 at mid-diastole. (4) Conclusion: Limiting CTA reading to distinct intervals or time points during the cardiac cycle has good specificity but lowers sensitivity. For a reliable diagnosis of HALT, data sets from a multiphase CTA covering the entire cardiac cycle should be analysed. A double reader approach would be desirable in further studies investigating HALT.

## 1. Introduction

Transcatheter aortic valve replacement (TAVR) is the current standard therapy for severe symptomatic aortic stenosis in patients with increased perioperative risk [1,2,3]. Recent studies described early hypo-attenuated leaflet thickening (HALT) or leaflet restriction in post-TAVR computed tomography angiography (CTA) with a prevalence ranging from 4% to 40% [4,5,6,7,8,9]. On the one hand, previous studies on medium-term outcomes demonstrated no impact on mortality or stroke [4,5,7,8,10,11]. On the other hand, HALT leads to valve deterioration, at least in some patients [6,8,12], and might be associated with valve degeneration [13].

Besides varying the time periods from intervention to CTA, the discrepancy in prevalence might be caused by using diverse CTA protocols covering either single parts or the entire cardiac cycle [4,5,10]. A standardized approach to CT data acquisition and interpretation may facilitate the comparability of findings across studies and may help to reduce the applied radiation dose. The Society of Cardiovascular Computed Tomography (SCCT) recommends full multiphase imaging covering the complete cardiac cycle [14]. As the TAVR procedure is progressively applied to younger and lower-risk patients, limiting cardiac cycle coverage may be desirable if there are concerns about radiation exposure. 

Therefore, we hypothesised that for the detection of HALT, investigating specific intervals and time points has comparable diagnostic results compared to the assessment of the entire cardiac cycle. In addition, we compared a single versus a two observer evaluation. 

## 2. Materials and Methods

Study population. This observational single-center cohort study was approved by the local institutional review board and complies with the Declaration of Helsinki. The study includes patients with post-TAVR-CTA between 2012 and 2017, when routine CTA was part of the clinical setup post TAVR in our hospital. Clinical reasons for not performing CTAs included renal insufficiency, frailty, and others [5]. Included valve types were the following commercially available balloon- or self-expandable prosthetic valves: SAPIEN XT, SAPIEN 3 (both Edwards Lifesciences, Irvine, CA, USA), CoreValve, Evolut R (both Medtronic, Minneapolis, MN, USA). 

HALT was defined as a hypo-attenuated thickening of one or more leaflets visible in at least two different projections and two different reconstruction time points with or without restricted motion, adapted to the definition of SCCT [12]. HALT is characterized as the thickening of the leaflets with a meniscal shape within the long-axis projection view at the center of the cusp, starting at the leaflet’s base [12]. The diagnosis was established by the consensus reading of two experienced observers (P.R. and T.K.) based on complete cardiac cycle analysis, considered the “gold standard”. 

CT scan protocol. The CTAs were performed on a 2nd generation dual-source CT scanner (Somatom Definition Flash, Siemens Healthcare, Forchheim, Germany). The post-TAVR-CTA-protocol comprised a contrast-enhanced retrospective ECG-gated CTA-data acquisition of the aortic root in a craniocaudal direction with a temporal resolution of 75 ms without ECG-gated dose modulation to cover the entire RR-interval. The Siemens “Care kV” and “Care Dose” options were used. 50 mL iodinated contrast agent (Imeron 400, Bracco, Konstanz, Germany) was administered at a flow rate of 4 mL/s. A bolus tracking technique with a region of interest placed in the left atrium was used. All CTA images were reconstructed at 50 ms steps throughout the cardiac cycle with a slice thickness of 1 mm, an increment of 0.8 mm, and a stent-specific reconstruction kernel (B46f). Those multiphase data sets were either fully reconstructed, at systole, at diastole, or at single systolic or diastolic time points to simulate image data of retrospective multiphase scan, a (pulsed) prospective scan, or a high pitch scan, respectively.

Image analysis. Image analysis was conducted with a dedicated post-processing workstation (Syngo Multimodality Workplace, Siemens Healthcare, Forchheim, Germany) using multiplanar reformations. Different phases of the cardiac cycle were evaluated regarding the capability to identify HALT by comparing them to the gold standard of HALT diagnosis. 

For the recent investigation, fifty anonymized patients with HALT plus fifty without HALT were selected using SPSS random selection. Two blinded observers (M.H. and M.S.) with CT-angiographic experience of more than 1000 CTAs re-evaluated the selected 100 data sets in blinded single and consensus reading. Systole and diastole from each patient were evaluated separately and randomly. Both observers were unaware of the number of patients with positive HALT-finding in the data set.

Mid-diastole was defined as 70 to 75% of the cardiac cycle according to the diastolic heart volume curve in the syngo. via software (Siemens Healthineers, Erlangen, Germany). End-systole was defined by the cardiac cycle curve. Single time points of mid-diastole and end-systole and separate phases of entire systole and diastole were anonymized and separately stored (P.R.). HALT in single time-point examination was diagnosed in only one reconstruction time point. The resultant four data sets were then separately evaluated (M.H. and M.S.) in random order. Each analysis was blinded from the results of the other cardiac cycle phase. First, all end-systolic and mid-diastolic data sets were analysed in separate sessions, followed by systole/diastole data sets again in separate sessions. 

Image quality was assessed on a scale of 0 to 3, with 0 reflecting the worst (non-diagnostic) image quality. One was defined as worse than standard quality. Two was standard quality with a clear contrast between prosthetic valve leaflets and blood volume. Three was better than average quality with perfect contrast between prosthetic valve leaflet and blood volume. Image quality in Table 1 was calculated as a rounded mean of reported image quality of both observers (M.S., M.H.). 

Statistical analysis. All statistical analyses were performed using SPSS-software (SPSS-version 23.0, Chicago, IL, USA). Continuous variables are reported as mean ± standard deviation, ordinal variables as median (interquartile range), and categorical data as numbers (percentages). Kolmogorov-Smirnov test was used to test for Gaussian distribution. For comparison between groups, unpaired Student’s *t*-test or Mann-Whitney U-test were used if appropriate. Differences in categorical variables were tested using the Pearson’s χ^2^-test or Fisher’s test depending on sample size. Sensitivity, specificity, negative, and positive predictive value (NPV and PPV), negative and positive likelihood ratios, accuracy, and Cohens Kappa were calculated to compare the diagnostic value of each cardiac time point/phase of CTA images. McNemar test was used to compare results of different readers and consensus reading. NPV and PPV for HALT-Finding were calculated with an estimated prevalence of 15.9% [13]. Paired Wilcoxon rank-sum was used to compare image quality between different phases. Variables with *p* < 0.10 at baseline as well as mitral valve regurgitation were assessed regarding their influence on HALT finding by logistic regression. Mitral valve regurgitation was added because of possible difficulties regarding the timing of image acquisition after contrast administration resulting in reduced image quality. As heart rate, ejection fraction, tricuspid/mitral valvular regurgitation (grade 0–4), and atrial fibrillation might have influenced image quality, the correlation between those factors and image quality or HALT diagnosis was assessed by using multiple regression.

## 3. Results

### 3.1. Patient Characteristics

Between 2012 and 2017, 761 patients with the above-described valves received a post-TAVR-CTA. In 105 of these patients, HALT was diagnosed. The randomly selected 100 CTA studies, 50 with and 50 without HALT, were acquired at a median of 5 days [IQR 4;5] after TAVR. Twenty patients with HALT revealed restricted motion, whereas, in the remaining patients, the motion was unaffected.

Baseline characteristics of the study population are shown in Table 1. The two groups were comparable regarding clinical baseline characteristics with a trend towards a lower prevalence of atrial fibrillation in patients with HALT (*p* = 0.083). Better image quality was found in the group with HALT (*p* = 0.010), whereas heart rate at the time of the CT was comparable between both groups (*p* = 0.440). Regarding echocardiographic parameters, there was a tendency towards fewer patients with moderate tricuspid regurgitation in the HALT group (*p* = 0.092). 

### 3.2. Sensitivity and Specificity of Various Cardiac Cycle Phases and Single Time Points

Sensitivity and specificity for the diagnosis of HALT were 84%/94% in the entire systole and 87%/92% in the entire diastole, respectively. Positive Likelihood ratios were higher than 10 in all parts of the cardiac cycle; however, negative likelihood ratios were above 0.10 (Table 2).

Regarding interpretation limited to single time points, sensitivity and specificity were 78%/95% at end-systole and 80%/94% at mid-diastole, respectively. End-systole had the highest positive predictive value (0.88) and positive likelihood ratio (36). The results of the HALT analysis of every single observer and consensus reading of each analysed cardiac cycle phase, along with each time point, are shown in Table 2.

### 3.3. Interobserver Reliability

Highest interobserver reliability was achieved in systole with a Cohens kappa of 0.715 (*p* < 0.001) for entire systole and 0.650 (*p* < 0.001) for end-systole. 

**Figure 1:**
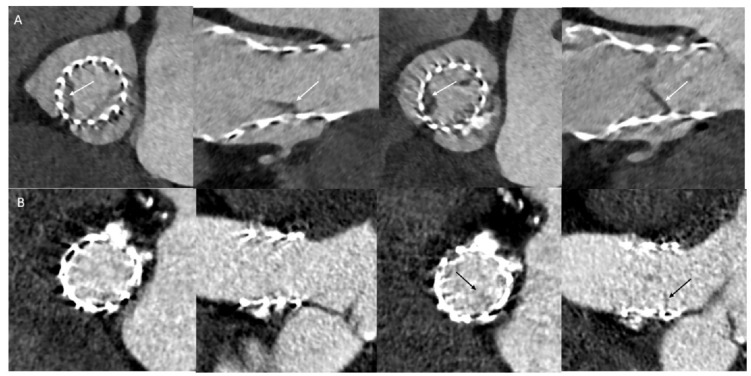
Examples of prostheses with Hypoattenuated Leaflet Thickening (HALT). (**A**) HALT (white arrows) depicted in systole (left) and diastole (right) (**B**) HALT (black arrows) missed in systole (left), but depicted in diastole (right).

In diastole, Cohens kappa for mid-diastole was 0.517 (*p* < 0.001) and 0.578 (*p* < 0.001) for entire diastole. Consensus reading in systole or diastole improved especially negative likelihood ratios (Table 2). Examples of HALT found in any cardiac phase or time point and of HALT missed in single observer investigation are shown in Figure 1.

### 3.4. Factors Associated with Inconsistent and Consistent HALT-Finding by Both Observers

In univariate analysis, atrial fibrillation (OR 2.364 [0.984;5.677], *p* = 0.054) and mitral regurgitation grading (OR 1.740 [0.910;3.325], *p* = 0.094) tended to be associated with inconsistent HALT findings by both single observers. Only observer reported image quality was associated with consistent HALT finding in univariate and multivariate analysis (OR 2.640 [1.355;5.131], *p* = 0.004 and OR 3.277 [1.459;7.358]; *p*= 0.004, respectively). Factors influencing image quality are summarized in Table 3.

## 4. Discussion

We report two main findings: First, reducing the CT protocol for diagnosing HALT to distinct phases or single time points of the cardiac cycle is associated with good specificity but lower sensitivity. Second, for a reliable diagnosis of HALT, acquiring images of the entire cardiac cycle and a double reader consensus approach is desirable.

Recent studies described HALT as a common phenomenon with a prevalence between 4–40% after TAVR [4,6,9,10]. Its long-term clinical relevance remains controversial, but HALT has been suspected to be associated with TIA, valve dysfunction, and valve degeneration [6,7,11,12,13,15]. So far, there is no evidence that routinely performed post-TAVR CTA would influence prognosis in cohorts of such old patients. As HALT leads to valve deterioration in some patients and as it can be treated by anticoagulation therapy, post-TAVR scans are of worth in clinical practice when there are echocardiographic signs of valve dysfunction [6,8,12,16]. Further, the TAVR procedure is expanding to low-risk cohorts and younger patients [1,17]. Especially, younger patients show a higher risk of valve degeneration after surgical valve replacement with bioprosthetic valves [7,12,18]. To avoid repeat valve interventions, especially in those with longer life expectancy, potential factors influencing durability should be investigated with reliable, standardized diagnostics with the lowest radiation exposure possible. From coronary CTA, we know that dose reduction is possible using different acquisition techniques. The effective dose of a high pitch spiral scan is about 13% of the dose of a multiphase retrospective scan, and the effective dose of a prospective (pulsed) scan is about 28% of a multiphase retrospective scan [19]. However, dose reduction should not lead to a decrease in diagnostic sensitivity. This is crucial for further investigation of the phenomenon of HALT and to draw confident conclusions regarding prevalence, natural history, and treatment strategies. In this line, the latest recommendations suggest image acquisition of the complete cardiac cycle in post-TAVR-CTA [14]. To the best of our knowledge, there is no published scientific evidence comparing any approaches. Furthermore, it is unclear whether a single observer approach, as commonly performed, is sufficient in this setting.

We report that limiting the CT scan evaluation to distinct phases of the cardiac cycle, albeit associated with good specificities and positive likelihood ratios, goes along with only moderate sensitivities. We, therefore, consider limiting CT scanning to distinct cardiac phases in general—in line with the current SCCT recommendations—as not advisable. In the present study, the gold standard diagnosis was established by consensus reading by two experienced observers. To us, this was the most accurate way to assess even small findings and might be the most appropriate way to diagnose HALT in clinical trials. However, even by defining consensus reading as the gold standard, this approach might not be immune against misclassification, and itself might have influenced the results. In daily routine, a single observer might be sufficient to detect HALT with concomitant leaflet restriction, as in those cases, thrombus volumes are often large. Therefore, some trials suggest using HALT with more than 50% leaflet involvement as study endpoints [20]. However, our data suggest that (even with experienced readers) a single observer approach may be insufficient if small thrombotic volumes are investigated. 

On the one hand, our data suggest as a rule of thumb: “If one observer sees a thrombus, there usually is one”. On the other hand, if there is only one observer or a CT scan with incomplete coverage of the cardiac cycle, HALT cannot be excluded. Therefore, future studies investigating the possibility of reducing radiation in post-TAVR CTAs to distinct cardiac phases by techniques like pulsing or even high pitch acquisition need to be planned with caution when any HALT extend is investigated. In clinical practice, extended masses of HALT might be more relevant. As patients with large HALT are rare, further (multicenter) studies investigating the sensitivity of limited CTA acquisition in patients with hemodynamic valve deterioration are desirable.

In our study, image quality was the only independent predictor for HALT. As expected, image quality in diastole was better than in systole [21,22]. Heart rate, in turn, was confirmed to influence image quality in diastole [23]. Other factors such as atrial fibrillation, ejection fraction, or valve regurgitation were not associated with image quality in any phase of the cardiac cycle. 

Computed tomographic findings of suspected leaflet thrombosis after TAVR vary substantially, resulting in several definitions, including hypo-attenuating leaflet thickening (HALT), hypo-attenuation affecting motion (HAM), and others [5,6,11,16]. In the current study, HALT is defined comprehensively as hypo-attenuated leaflet thickening independent of motion-related factors or the number of reconstruction time points, thereby covering both HALT and HAM [16]. 

## 5. Conclusions

To obtain high sensitivity for the diagnosis of early HALT after TAVR, a CT protocol covering the entire cardiac cycle as suggested in current recommendations is advisable. A double reader approach would be desirable in further studies investigating HALT. 

## 6. Limitations

Our study is small, and though issued from a center with long-standing experience in cardiac imaging, results require external validation. We did not perform repeat CTAs using different image acquisition techniques in each patient (retrospective full multiphase vs. prospective pulsed imaging vs. high pitch imaging), but used retrospective multiphase scans as the basis for comparison. Therefore, direct comparison of radiation exposure is not possible. Issues of standardizing diagnostics such as the timing of CTA concerning the TAVR procedure have to be addressed in further studies. Finally, the gold standard for diagnosing HALT was based on a full cardiac cycle CTA protocol with two readers. Although this appears adequate compared to less extensive protocols, alternate modalities may prove more accurate in the future. Upcoming photon-counting CTs might reduce metal artifacts to facilitate automated algorithms for HALT detection. We did not differ between various extents of HALT, as only a few patients showed large findings directly after TAVR. This should be addressed in further studies investigating patients who qualify for symptomatic hemodynamic valve deterioration.

## Figures and Tables

**Table 1:** Baseline characteristics of patients without and with HALT.

		All Patients (*n* = 100)	Without HALT (*n* = 50)	With HALT (*n* = 50)	*p*-Value
Age		81.5 ± 5.5	80.1 ± 5.4	82.2 ± 5.5	0.185
Gender					
Male		37 (37.0)	16 (32.0)	21 (42.0)	0.400
Female		63 (63.0)	34 (68.0)	29 (58.0)	
BMI	(kg/m²)	27.1 ± 4.3	27.3 ± 4.7	27.0 ± 3.8	0.809
Creatinine-Clearance	(mL/min)	54.7 ± 17.3	55.5 ± 18.6	53.9 ± 16.1	0.648
LV-EF	(%)	50.0 (8.3)	51.5(8.1)	49.1 (8.4)	0.162
Atrial fibrillation		31 (31.0)	20 (40.0)	11 (22.0)	0.083
Mitral regurgitation	None/mild	76 (76.0)	37 (74.0)	39 (78.0)	0.591
	Moderate	20 (20.0)	10 (20.0)	10 (20.0)	
	Severe	4 (4.0)	3 (6.0)	1 (2.0)	
Tricuspid regurgitation	None/mild	90 (90)	42 (84)	48 (96)	0.092
	Moderate	10 (10)	8(16)	2(4)	
Heart rate at the time of CT		54.7 ± 17.3	55.8 ± 18.6	53.9 ± 16.1	0.440
Tube voltage (kV)		102.8 ± 9.9	103.2 ± 10.2	100.0 ± 9.9	0.835
Tube current (mAs)		307.7 ± 51.1	302.0 ± 49.4	307.7 ± 52.9	0.605
Effective Dose (mSv)		9.8 ± 5.1	10.2 ± 10.2	9.5 ± 5.1	0.336
Prosthesis size	Small (23 mm)	33 (34.7)	18 (36.0)	15 (33.3)	0.867
	Medium (25–27 mm)	39 (41.1)	21 (42.0)	18 (40.0)	
	Large (29–31 mm)	23 (24.2)	11 (22.0)	12 (26.7)	
Prosthesis type	S3/XT	86 (86.0)	43 (86.0)	43(86.0)	1.000
	CoreValve/EvoluteR	14 (14.0)	7 (14.0)	7 (14.0)	
PVL at time of CTA (TTE)	None/Trace	81 (81.0)	40 (80.0)	41 (81.0)	1.000
	Mild	19 (19.0)	10 (20.0)	9 (18.0)	
	Moderate	0	0	0	
Visual Image Quality	0	1 (1.0)	1 (2.0)	0 (0.0)	0.010
	1	10 (10.0)	6 (12.0)	4 (8.0)	
	2	51 (51.0)	32 (64.0)	19 (38.0)	
	3	38 (38.0)	11 (22.0)	27 (54.0)	

HALT: hypo-attenuated leaflet thickening. Values are mean ± SD, median [interquartile range], or *n* (%). BMI= body mass index. LV-EF: Left ventricular ejection fraction. PVL: Paravalvular leakage. TTE: Transthoracic echocardiogram. mAs: milliampere-second. kV: kilovoltage. Image quality was graded in consensus as follows: 0 = very poor image quality, 1 = poor, 2 = good, 3 = very good.

**Table 2:** Comparison of single observer diagnosis of HALT in different phases of the cardiac cycle with full cardiac cycle consensus reading.

	Diastole						Systole					
	Observer A		Observer B				Observer A		Observer B			
	Mid-Diastole	Entire Diastole	Mid-Diastole	Entire Diastole	Consensus Mid-Diastole	Consensus Entire Diastole	End-Systole	Entire Systole	End-Systole	Entire Systole	Consensus End-Systole	Consensus Entire Systole
True positive	38	42	32	42	38	43	36	42	32	38	36	41
True negative	46	45	47	47	49	47	47	46	49	47	49	46
False positive	4	5	3	3	1	3	3	4	1	3	1	4
False negative	12	8	18	8	12	12	14	8	18	12	14	9
% Accuracy	0.84	0.87	0.79	0.89	0.87	0.90	0.83	0.88	0.81	0.85	0.85	0.87
% Sensitivity	0.76	0.84	0.64	0.84	0.80	0.87	0.72	0.84	0.64	0.76	0.78	0.84
% Specificity	0.92	0.90	0.94	0.94	0.94	0.92	0.94	0.28	0.98	0.94	0.95	0.94
PPV	0.64	0.61	0.67	0.73	0.71	0.67	0.69	0.66	0.86	0.71	0.88	0.66
NPV	0.63	0.59	0.67	0.65	0.65	0.61	0.66	0.62	0.73	0.66	0.73	0.62
PLR	9.5	8.2	10.7	14.0	13.3	10.7	12.0	10.2	32	12.7	36	10.3
NLR	0.26	0.18	0.38	0.17	0.21	0.14	0.30	0.18	0.37	0.26	0.24	0.17

HALT = hypo-attenuated leaflet thickening, PPV = positive predictive value, NPV = negative predictive value, PLR = positive likelihood ratio, NLR = negative likelihood ratio.

**Table 3:** Linear regression of factors influencing image quality.

	Heartrate		Ejection Fraction		Mitral Regurgitation		Tricuspid Regurgitation	
	Standardized Beta	*p*	Standardized Beta	*p*	Standardized Beta	*p*	Standardized Beta	*p*
Mid-diastole	−0.40	<0.001	−0.12	0.22	0.13	0.194	0.11	0.28
entire diastole	−0.32	0.002	−0.10	0.32	0.14	0.164	0.075	0.47
end-systole	−0.07	0.54	−0.127	0.21	0.043	0.67	0.08	0.44
entire systole	−0.06	0.55	0.73	0.47	0.02	0.81	0.07	0.49

## Data Availability

Transferring data to third parties is not included in the ethics statement. Please contact the corresponding author for submitting a request to the ethics committee, if desired.
